# Fecal Incontinence Outcomes Following Transvaginal Posterior Vaginal Wall Repair

**DOI:** 10.1007/s00192-025-06096-z

**Published:** 2025-03-22

**Authors:** Jersey B. Burns, Amr El Haraki, Jesseca Crawford, Candace Y. Parker-Autry

**Affiliations:** 1 Department of Obstetrics and Gynecology, Atrium Health Wake Forest Baptist, Winston-Salem, NC USA; 2 Department of Urology, Atrium Health Wake Forest Baptist, 1 Medical Center Boulevard, Winston-Salem, NC 27157 USA

**Keywords:** Anorectal manometry, Fecal incontinence, Posterior Repair, Rectocele, Sphincteroplasty

## Abstract

**Introduction and Hypothesis:**

Knowledge regarding rates of improvement of fecal incontinence (FI) after repair of posterior compartment prolapse is limited. We aimed to estimate the rate of resolution or improvement of fecal incontinence postoperatively following transvaginal posterior compartment repair.

**Methods:**

This was a retrospective cohort study including patients with diagnosis of fecal incontinence who underwent transvaginal posterior repair at a single academic institution between 1/2016 and 1/2022. Patients who underwent concomitant anal sphincteroplasty served as controls. The primary outcome was resolution of FI symptoms within 6-weeks postoperatively. Secondary outcomes included improvement of FI symptoms and preoperative anorectal manometry characteristics. Univariate and bivariate analysis were performed to describe and compare outcomes between groups with multivariable regression performed to address potential confounders.

**Results:**

Of 179 patients included, 91 had posterior repair alone, while 88 had concomitant anal sphincteroplasty. Demographic and clinical characteristics were similar between groups. Overall, 143 (80%) patients did not report any FI symptoms at their 6-week postoperative visit. An additional 28 (16%) reported improvement in FI symptoms. Among patients who underwent posterior repair alone, 76 (84%) had resolution of their FI compared to 67 (76%) in patients with concomitant anal sphincteroplasty (*P* = 0.6); 71 patients underwent anorectal manometry preoperatively. There were no significant differences in mean average resting pressures or mean maximum squeeze pressures between groups.

**Conclusions:**

Posterior compartment repair resulted in resolution or improvement of fecal incontinence symptoms within 6-weeks postoperatively. The mechanism for FI symptoms in women with rectoceles may be independent of the anal sphincter complex.

## Introduction

Posterior vaginal wall prolapse may refer to rectocele, enterocele, and/or sigmoidocele and is prevalent among women over the age of 50 resulting from disruption of the support to the vaginal apex, mid-vaginal endopelvic fascia, and the perineal body [1]. Up to 30–50% of women may be symptomatic from posterior vaginal wall prolapse and may present clinically with symptoms of vaginal bulge, defecatory dysfunction featuring digital splinting and incomplete rectal evacuation, and fecal or anal incontinence. Fecal incontinence (FI) occurs in 20% of women with posterior vaginal wall prolapse [2, 3].

Posterior vaginal wall prolapse may result in stool trapping, weakness of the anal sphincter complex with perineal body attenuation, deficiencies in anorectal sampling allowing for greater rectal capacity and stool burden, as well as a decreased anorectal angle from pelvic floor weakness [4, 5]. We hypothesize the mechanism for fecal incontinence in women with posterior vaginal wall prolapse relate to attenuation of the perineal body and stool trapping in the distal rectal vault that results in “overflow” fecal incontinence. On the basis of this and prior reported data, posterior vaginal wall repair and perineorrhaphy, independent of the anal sphincter, may improve fecal incontinence symptoms. Further, we hypothesize that anorectal manometry evaluation of anorectal function in the presence of rectocele may evidence predictors of resolution of FI when performed preoperatively.

To date, clinical associations between presence of posterior vaginal wall prolapse and fecal incontinence is limited. We have executed a retrospective cohort study using prospectively collected data to primarily examine the impact of rectocele repair on fecal incontinence symptoms present in women with symptomatic pelvic organ prolapse. Secondarily, we aimed to clinically characterize anorectal function of women with posterior vaginal wall prolapse.

## Materials and Methods

This is a retrospective cohort of a convenience sample of patients with a diagnosis of fecal incontinence who underwent transvaginal native tissue posterior vaginal wall repair for symptomatic posterior vaginal wall prolapse and their fecal incontinence symptoms. Symptoms of prolapse included vaginal bulge and difficulty with rectal or bladder evacuation at a single academic institution between 1/2016 and 1/2022. In addition, electronic medical record (EMR) abstraction confirmed the presence of fecal incontinence symptoms based on the initial history of present illness interview. Procedures were performed by resident or fellow trainees supervised by two academic urogynecology attending surgeons. Patients were identified by the ICD-10 diagnosis code for fecal incontinence (R15 – fecal incontinence, R15.1—fecal smearing, R15.0—incomplete defecation, R15.9—full incontinence) with an associated CPT code for rectocele repair (57,260 – Anterior and posterior repair, 57,265 – anterior and posterior repair with enterocele, 57,250 – posterior repair, 45,560 – repair of rectocele). Patients were excluded if they had missing baseline or post operative fecal incontinence symptom data, diagnoses of flatal incontinence or fecal urgency alone, or if they did not have fecal incontinence symptoms at the preoperative visit as determined by review of patient reported history. Patients with both flatal and fecal incontinence together were included. Patients were excluded if they did not present for a postoperative visit.

Patients with concomitant anal sphincteroplasty were included as controls. Performance of sphincteroplasty was determined by electronic medical record review of the operative note. The decision to perform anal sphincteroplasty concomitantly was a shared decision after preoperative patient counseling with the aim to correct an anatomic defect in the anal sphincter confirmed by endoanal ultrasound. Two researchers abstracted key demographic and clinical data from the electronic medical record. Patient demographics included age, parity, BMI, menopausal status, current tobacco use, and diabetes diagnosis. Clinical characteristics collected included concomitant surgical procedures, duration and type of fecal incontinence, presence of constipation, and need for manual disimpaction maneuvers (splinting). An audit of data collection was performed on 10 charts prior to data analysis by an independent study team member. Fecal incontinence symptoms were determined at baseline interview and at postoperative evaluation taking place between postoperative weeks 4–6 through a single question, “Do you ever have any bowel accidents?”. The primary outcome was percent of patient reported resolution of FI symptoms at 6-weeks postoperatively. If bowel accidents were reported, symptoms were stratified into same, better, or worse based on patient description at postoperative visit compared to preoperative visit. Improvement of FI symptoms were examined secondarily.

Anorectal physiology testing data were abstracted when available to include anorectal manometry, balloon expulsion testing, and endoanal ultrasound. Anorectal manometry included anal canal length (cm), mean average resting pressure (mmHg), mean maximum squeeze pressure (mmHg), threshold of first defecatory urge (mL), maximum tolerable volume (mL), presence of recto-anal inhibitory reflex (RAIR), and volume to illicit RAIR (mL). The balloon expulsion test was performed at the volume of a 50 mL balloon seated on a toilet with electromyelogram to identify presence of dyssynergia. All normative values used were predetermined by Laborie^®^ anorectal manometry software. Endoanal ultrasound was performed using the standardize approach with the BK flexFocus 500.

For the data analysis, the primary outcome was the presence of fecal incontinence symptoms at the early postoperative assessment visit. Categorical variables were analyzed using χ^2^ tests and Fisher exact tests. Continuous variables were analyzed using *t* test. Univariate and bivariate analysis were performed to describe and compare outcomes between groups. Statistical analysis was performed using R (RStudio, PBC, Boston, USA). *P* < 0.05 was considered to be statistically significant. A post-hoc power analysis was performed.

## Results

**Fig. 1:**
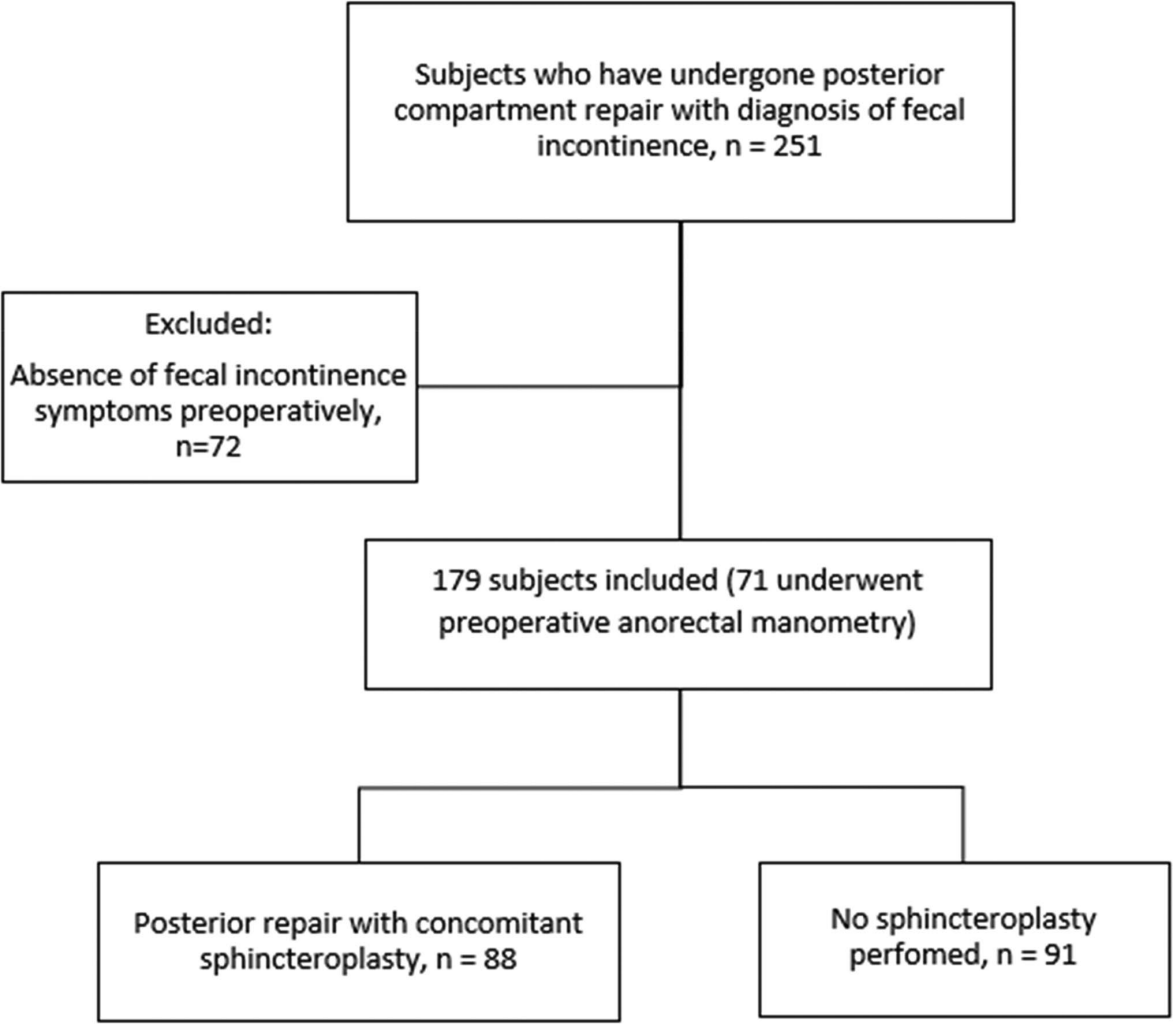
Participant flow chart

Two-hundred forty-six charts were reviewed to identify a total of 179 patients who underwent transvaginal posterior vaginal wall repair and had fecal incontinence symptoms preoperatively. Of those, 91 had vaginal wall repair alone, while 88 patients had posterior vaginal wall prolapse with anal sphincter defects and underwent concomitant anal sphincteroplasty (controls) (Fig. 1). None of the 37 participants excluded had missing data. Demographic characteristics were similar between groups (Table 1). The mean age was 61 ± 13 years, and mean BMI was 28.1 ± 7.4 kg/m^2^. The majority (91%) of our cohort were non-Hispanic White and 9% (*n* = 16) were Black with the remainder of the cohort identifying as other race or non-reporting. The median stage of pelvic organ prolapse was Stage II for both groups (*P* = 0.6) (Table 1).

**Table 1:** Clinical and demographic characteristics of the cohort based on type of surgical intervention

Patient characteristics	Posterior repair (*n* = 91)	Posterior repair with sphincteroplasty (*n* = 88)	*P *value
Age at time of surgery	60.9 ± 13.9	60.9 ± 13.5	0.90
Number of delivered children (parity)	3 (2.00–3.00)	2 (2–3)	0.64
BMI (kg/m^2^)	30.27 ± 8.79	29.03 ± 5.75	0.26
Postmenopausal	79 (86.8%)	70 (79.5%)	0.63
Tobacco use, current	9 (9.89%)	5 (5.68%)	0.29
Diabetes mellitus	17 (18.7%)	9 (10.2%)	0.10
Concomitant surgical procedure			
Anterior repair	18 (19.8%)	22 (25%)	0.41
Apical repair	49 (53.8%)	48 (54.5%)	0.93
SSLF	16	19	
USLS	19	22	
SCP	14	7	
Perineorrhaphy	52 (57.1%)	38 (43.2%)	0.06
Stress incontinence procedure	41 (45.1%)	33 (3.5%)	0.28
Clinical presentation			
Duration of fecal incontinence	*N* = 32	*N* = 36	0.94
Less than 1 year	6	7	
Greater than 1 year	26	29	
Type of fecal incontinence			
Flatus	33 (36.3%)	20 (22.7%)	0.05
Liquid stool	42 (46.2%)	50 (56.8%)	0.15
Solid stool	34 (37.4%)	35 (39.8%)	0.74
Insensible	22 (24.2%)	10 (11.4%)	0.03
Urgency	45 (48.5%)	36 (40.9%)	0.25
Chronic constipation	19 (20.9%)	24 (27.3%)	0.32
Manual disimpaction maneuvers	17 (18.7%)	20 (22.7%)	0.50
Bulge sensation	65 (71.4%)	61 (69.3%)	0.76
Dyspareunia	18 (19.8%)	18 (20.5%)	0.91
Physical exam			
POPQ stage			
Ap	0 (−1.00 to 0)	0 (−1.00 to 1.00)	0.26
Bp	−1.00 (−2.00 to 0)	−.05 (−1.00 to 1.00)	0.17
C	−4.50 (−6.00 to 2.00)	−4.00 (−6.00 to −0.50)	0.42
TVL	9.00 (8.00–10.00)	9.00 (8.00–10.00)	0.29
Gh	4.00 (3.50–5.00)	4.00 (3.50–5.00)	0.25
Stage of pelvic organ prolapse	2.00 (2.00–3.00)	2.00 (2.00–3.00)	0.60

Overall, 143 (80%) patients did not report any fecal incontinence by their 6-week postoperative visit. Subjective postoperative fecal incontinence symptoms resolved with similar rates in both groups (84% in the posterior repair group and 76% in the sphincteroplasty group, *P* = 0.6). An additional 15 (16%) and 21 (24%) of patients (*P* = 0.22) reported improvement, though not resolution, in their preoperative fecal incontinence symptoms in the two groups (Table 2). Of the 36 participants with persistent FI symptoms postoperatively, 15/91 had posterior vaginal wall repair alone and 21/88 had concomitant anal sphincter repair. Surgical site infection as defined within 30 days of surgery was treated in six patients in the concomitant sphincteroplasty as compared to one patient in the posterior repair group. Post hoc power analysis revealed 38% power to detect a difference between these two cohorts with an alpha of 0.10.

**Table 2:** Subjective outcomes of anorectal function determined by 6 weeks postoperatively

	Posterior repair (*n* = 91)	Posterior repair with sphincteroplasty (*n* = 88)	*P* value
No reported fecal incontinence (at 4–6 week postoperative visit)	76 (84%)	67 (76%)	0.6
Fecal incontinence reported (at 4–6 week postoperative visit)	15 (16%)	21 (24%)	0.22
Same	1 (1.1%)	1 (1.1%)	
Better	11 (12%)	18 (20%)	
Worse	2 (2.2%)	2 (2.3%)	
Constipation	8 (8.8%)	8 (9.1%)	0.33
Same	3	3	
Better	1	1	
Worse	4	4	
Manual disimpaction maneuvers	1 (1.1%)	1 (1.1%)	0.99
Same	1	N/A	
Better	N/A	N/A	
Worse	N/A	N/A	
Bulge symptoms	0 (0%)	0 (0%)	
Same	N/A	N/A	
Better	N/A	N/A	
Worse	N/A	N/A	
Postoperative surgical site infection within 30 days	1 (1%)	6 (7%)	
Need for reoperation for surgical complication within 30 days	2 (2%)	1 (1%)	0.58

Seventy-one of the 143 patients underwent anorectal manometry preoperatively for the indication of fecal incontinence. For the overall cohort, the mean average resting pressure was normotensive at 53.8 ± 22 mmHg and the mean maximum squeeze pressure was hypotensive at 81.7 ± 37 mmHg. Average canal length was shortened to 2.87 ± 1.16.

## Discussion

In this cohort of 179 women with symptomatic posterior vaginal wall prolapse and fecal incontinence symptoms, fecal incontinence symptoms were resolved and significantly improved in the majority during the early postoperative period. This observed decrease in FI symptoms was comparable to the decrease observed in women undergoing posterior repair with anal sphincteroplasty. This supports the notion that fecal incontinence symptoms can improve with transvaginal posterior compartment repair alone. Other studies have shown improvement with transperineal repair that involved concomitant sphincteroplasty [7]. The findings in this study suggest a mechanism for fecal incontinence independent of sphincter disruption in patients with posterior compartment prolapse.

Clinical associations between rectocele and fecal incontinence symptoms vary and prior studies have been limited to small observational studies. A previous prospective trial found improvement in bowel symptoms, including fecal incontinence 1 year following posterior compartment repair [6]. Young and Robles described their success with transperineal rectocele repair with concomitant anal sphincter repair with excellent success at 6 months [7]. They experienced wound dehiscence in 13% of patients. This data aligns with our observations of resolution of fecal incontinence after posterior repair with and without anal sphincter repair. It is notable that our infection rate was much lower at 7%. On the contrary, Van Dam and colleagues describe risk of developing fecal incontinence in their observational study of 89 women undergoing transvaginal/transanal rectocele repair for obstructed defecation [8]. Our data supports that transvaginal approach to rectocele repair decreases fecal incontinence.

Integration of anorectal physiology testing is recommended in the evaluation of women with fecal incontinence. Broens and colleagues conducted magnetic resonance defecography and anorectal function testing during defecation on 32 women with suspected rectocele to understand functional contributors to rectocele development or enlargement [9]. They observed in some women an increase in anal sphincter pressure just before defecation that was positively correlated with straining maneuvers, defecation blockage, and rectocele size. Their observations suggest a clinically important observation of anal sphincter dyssynergia in some women with symptoms of fecal incontinence, defecatory dysfunction, and suspected rectocele, thus providing evidence for preoperative anorectal manometry in women with these clinical features. In the presence of anal sphincter dyssynergia, anorectal biofeedback would be the primary treatment in leu of correcting the anatomic distortion of rectocele. Our findings also suggest that in 20% of women with rectocele, dyssynergia may be present and untreated by surgical intervention.

Our study is strengthened by its large convenience sample of 179 symptomatic patients with confirmed posterior vaginal wall prolapse and fecal incontinence. Owing to the sample being of patients evaluated by board-certified Urogynecology faculty, associated symptoms of fecal incontinence, defecatory dysfunction, and prolapse were well characterized. Further, endoanal ultrasound was performed using a standardized technique by trained faculty providers to confirm the presence of anal sphincter injury. Our final strength is in the ability to compare the posterior repair group to a control group of posterior repair with anal sphincteroplasty as there is strong plausibility that the latter would have significant reduction in fecal incontinence symptoms with addition of sphincter repair.

There are significant study limitations that require these observations to be confirmed with a prospective study. The most important to consider is the lack of use of validated questionnaires that provide symptom severity data before or after intervention. However, the presence or absence of fecal incontinence does not require standardization to assessing. We recognized that the change in fecal incontinence episodes and severity is best elicited with standardized questionnaires. The retrospective nature of data analysis introduces the possibility of misclassification bias. In addition, confounders, including the possibility of patients having constipation at short-interval postoperative visit which may be masking baseline FI symptoms, were not elicited in the retrospective data collection. Lastly, the data should be interpreted to reflect short-term follow-up as long-term follow-up was not available for this study but could be addressed in future, prospective studies. While rectal prolapse, a risk factor for fecal incontinence symptoms, was not specifically abstracted, within our practice, rectal prolapse is screened for, and we do not perform posterior vaginal wall prolapse repair in the setting of rectal prolapse. Finally, as demonstrated by the case series of De Robles and Young, it is possible that those included in the posterior repair only group may have had perineorrhaphy procedures that included plication of the external anal sphincter, thus making the comparator group less robust [9]. To this point, we suggest focusing on the major strength of this study which is the pre- and postoperative assessment of the presence of fecal incontinence observed adequately in this design.

In conclusion, women with fecal incontinence and posterior vaginal wall prolapse may experience a significant reduction in their fecal incontinence symptoms after repair. Future studies are needed to evaluate the role of anorectal physiology measures as potential predictors of fecal incontinence or defecatory dysfunction resolution to best understand the mechanisms underlying fecal incontinence in the setting of posterior vaginal wall prolapse.

## Data Availability

Deidentified data can be made available with institutional approval upon request.

## Declarations

### Prior Presentations

1. International Continence Society, September 2023, Toronto, Canada.

2. American Urogynecologic Society, October 2023, Portland, Oregon, United States.

## References

[1] Aubert M, Mege D, Le Huu NR, Meurette G, Sielezneff I. Surgical management of the rectocele - an update. J Visc Surg. 2021;158(2):145–57. 10.1016/j.jviscsurg.2020.10.001. 33495108 10.1016/j.jviscsurg.2020.10.001

[2] Meschia M, Buonaguidi A, Pifarotti P, Somigliana E, Spennacchio M, Amicarelli F. Prevalence of anal incontinence in women with symptoms of urinary incontinence and genital prolapse. Obstet Gynecol. 2002;100(4):719–23. 10.1016/s0029-. 12383540 10.1016/s0029-7844(02)02215-9

[3] Lee PY, Steele SR. Complete pelvic floor repair in treating fecal incontinence. Clin Colon Rectal Surg. 2005;18(1):55–9. 10.1055/s-2005-864082. 20011341 10.1055/s-2005-864082 PMC2780130

[4] Hannaway CD, Hull TL. Fecal incontinence. Obstet Gynecol Clin North Am. 2008;35(2):249–69, viii. 10.1016/j.ogc.2008.03.005. 10.1016/j.ogc.2008.03.005 18486840

[5] Rao SS. Pathophysiology of adult fecal incontinence. Gastroenterology. 2004;126(1 Suppl 1):S14-22. 10.1053/j.gastro.2003.10.013. 14978634 10.1053/j.gastro.2003.10.013

[6] Gustilo-Ashby AM, Paraiso MF, Jelovsek JE, Walters MD, Barber MD. Bowel symptoms 1 year after surgery for prolapse: further analysis of a randomized trial of rectocele repair. Am J Obstet Gynecol. 2007;197(1):76.e1-5. 10.1016/j.ajog.2007.02.045. 17618766 10.1016/j.ajog.2007.02.045

[7] De Robles MS, Young CJ. Transperineal rectocele repair is ideal for patients presenting with fecal incontinence. Ann Coloproctol. 2022;38(5):376–9. 10.3393/ac.2021.00157.0022. 34663063 10.3393/ac.2021.00157.0022 PMC9650349

[8] Van Dam JH, Huisman WM, Hop WC, Schouten WR. Fecal continence after rectocele repair: a prospective study. Int J Colorectal Dis. 2000;15(1):54–7. 10.1007/s003840050008. 10766092 10.1007/s003840050008

[9] Sun G, de Haas RJ, Trzpis M, Broens PMA. A possible physiological mechanism of rectocele formation in women. Abdom Radiol (NY). 2023;48(4):1203–14. 10.1007/s00261-023-03807-2. 36745205 10.1007/s00261-023-03807-2 PMC10115871

